# Electrochemical energy storage performance of 2D nanoarchitectured hybrid materials

**DOI:** 10.1038/s41467-021-23819-0

**Published:** 2021-06-11

**Authors:** Jie Wang, Victor Malgras, Yoshiyuki Sugahara, Yusuke Yamauchi

**Affiliations:** 1 JST-ERATO Yamauchi Materials Space-Tectonics Project, Kagami Memorial Research Institute for Materials Science and Technology, Waseda University, Shinjuku-ku, Tokyo Japan; 2 JST-ERATO Yamauchi Materials Space-Tectonics Project, International Center for Materials Nanoarchitechtonics (WPI-MANA), and International Center for Young Scientists (ICYS), National Institute for Materials Science (NIMS), Tsukuba, Ibaraki Japan; 3 Faculty of Science and Engineering, Waseda University, Shinjuku, Tokyo Japan; 4 Australian Institute for Bioengineering and Nanotechnology (AIBN), The University of Queensland, Brisbane, QLD Australia

**Keywords:** Energy, Materials for energy and catalysis

## Abstract

The fast-growing interest for two-dimensional (2D) nanomaterials is undermined by their natural restacking tendency, which severely limits their practical application. Novel porous heterostructures that coordinate 2D nanosheets with monolayered mesoporous scaffolds offer an opportunity to greatly expand the library of advanced materials suitable for electrochemical energy storage technologies.

## Limitations of 2D materials for electrochemical energy storage

Since graphene was first experimentally isolated in 2004, many other two-dimensional (2D) materials (including nanosheet-like structures), such as transition metal oxides, dichalcogenides, and transition metal carbides/nitrides (MXenes), have been increasingly studied.^1,2^ Their unparalleled properties (e.g., electric conductivity, redox potential, and high packing density) and surface chemistry (e.g., electrocatalytic activity, chemical inertness, and polarity) have been widely investigated for their potential roles in electrochemical energy storage applications.^3^ However, none of these 2D materials can offer all the properties required to achieve the maximum energy/power density and cycling life. For example, the theoretical gravimetric capacitance of graphene can reach over 550 F g^–1^,^4^ while the state-of-the-art capacity of graphene-based supercapacitors in nonaqueous electrolytes is less than 250 F g^–1^.^5^ The main experimental limitation of graphene electrodes is the reduced ion-accessible surface area due to restacking. Heterostructures built by stacking different 2D materials in heterolayered architectures have enabled a new paradigm in 2D materials science.^6^ Not only do these materials combine the attributes and functionalities of the individual building blocks, but the resulting synergistic performance has triggered increasing fascination in the scientific community. However, in regard to electrochemical applications, chemically synthesized 2D material-based heterostructures still suffer from an intrinsic restacking tendency, which limits ion transport and reduces accessibility to the surface.^6^ Thus, it is necessary to engineer the interlayer space of 2D materials to promote the charge-transport capability and enhance their electrolyte-accessible surface area to reach their full charge-storage potential.

The hybridization of 2D nanosheets with other low-dimensional materials, such as nanotubes and nanoparticles, can generate additional channels for ion transport within the interlayer space.^7,8^ It is difficult to obtain the uniform dispersion of components simply by mixing because agglomeration is unavoidable. In addition, the tortuous space created by assembling randomly dispersed low-dimensional materials does not permit ions to diffuse efficiently. A large quantity of electrolyte is needed to fill all the void spaces to guarantee proper ion transport. Two-dimensional hybrids can be developed further to obtain an architecture that can enable access to nanospaces and enhance ion transfer.

## 2D porous heterostructures for enhancing ion transport

**Fig. 1:**
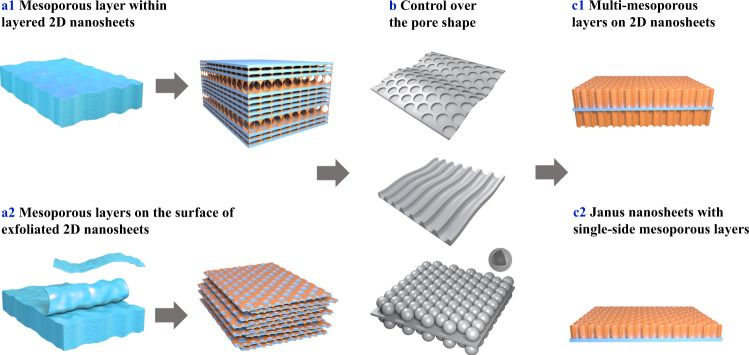
Present and future prospects of 2D porous heterostructures. Construction of 2D porous heterostructures from (**a**1) layered 2D nanosheets and (**a**2) exfoliated 2D nanosheets. **b** Control over the pore shape of the mesoporous layer. Future design of a 2D porous heterostructure (**c**1) with multiple mesoporous layers and (**c**2) by using Janus nanosheets.

Recently, a class of 2D porous heterostructures in which an ultrathin 2D material is sandwiched between two mesoporous monolayers (Fig. 1) has emerged as a research horizon for supercapacitors and secondary batteries.^9–15^ When used in these energy storage applications, mesoporous monolayers with uniform and tunable pore sizes take on several critical roles: 1) lowering the transfer resistance of the reactants (products) towards (from) active sites and allowing the electrolyte to fully access to the 2D nanosheets by providing interlayer spacing between 2D nanosheets, thus leading to an improvement of capacitance and power density in supercapacitors; 2) introducing additional active sites, thus partially contributing to increase the capacity and energy density in secondary batteries; and 3) providing the ability to host guest functional particles, promoting an enhanced performance for energy storage applications such as sulfur hosting in lithium–sulfur batteries. Basic procedures for the fabrication of 2D mesoporous heterostructures include the assembly of 2D nanosheets and organic molecule/block polymer composite micelles, polymerization of organic molecules, and removal of block polymers.^16^

Our group developed an evaporation-induced self-assembly (EISA) strategy to synthesize a layer-by-layer heterostructure by introducing ordered mesoporous carbon (OMC) layers within the interlayer space of stacked Ti_3_C_2_T_*x*_ (T = F, OH, *etc*.) nanosheets (Fig. 1a1).^9^ Upon gradual evaporation of the solvent (ethanol), the Pluronic F127 micelle@resol self-assembles between the Ti_3_C_2_T_*x*_ nanosheets to form a closely packed interlayer, which is then converted to a Ti_3_C_2_T_*x*_-OMC heterostructure after thermal treatment in an inert atmosphere. When integrated into supercapacitors, these Ti_3_C_2_T_*x*_-OMC electrodes exhibit a much higher gravimetric capacitance and better rate capability than pristine Ti_3_C_2_T_*x*_ electrodes. Therefore, although the density of Ti_3_C_2_T_*x*_-OMC (3.1 g cm^–3^) is slightly lower than that of Ti_3_C_2_T_*x*_ (3.4 g cm^–3^), its volumetric capacitance is noticeably higher (198 *vs* 177 F cm^–3^). Electrodes obtained by etching Ti from Ti_3_C_2_T_*x*_-OMC and Ti_3_C_2_T_*x*_ through high-temperature chlorination exhibit surface area-normalized capacitances of 24 and 15 μF cm^–2^, respectively, highlighting the benefits of the additional porosity brought by the interstitial OMC layers. Notably, Ti-etched Ti_3_C_2_T_*x*_-OMC electrodes show only a slight decrease in capacitance upon increasing the areal density from 5 to 12 mg cm^–2^, suggesting a strong potential for fabricating thick electrodes for commercial devices. The porous heterostructure promotes mass transport; enhances the accessibility of electroactive sites to ions, leading to an increased capacitance and rate capability; and facilitates electron transfer in the *c*-direction, thus improving electrical conductivity.

Solution-based synthesis is a more widely applied method to obtain organic molecule/micelle composites assembled with exfoliated 2D nanosheets. We fabricated a sandwich-like heterostructure of mesoporous carbon@Ti_3_C_2_T_*x*_@mesoporous carbon (C@Ti_3_C_2_T_*x*_@C) involving the self-assembly of F127/melamine-formaldehyde resin (F127/MF) composite micelles on the surface of exfoliated Ti_3_C_2_T_*x*_ nanosheets (Fig. 1a2).^10^ Liquid phase chemistry causes the F127/MF composite micelles to adhere to the surface of the Ti_3_C_2_T_*x*_ nanosheets in a closely packed fashion and to cross-link further into a thin shell. When the carbonized, porous heterostructured C@Ti_3_C_2_T_*x*_@C composite is used as a sulfur host for lithium–sulfur batteries, the C@Ti_3_C_2_T_*x*_@C/S cathode manifests a higher capacity and considerably improved rate and cycling performance compared with pure Ti_3_C_2_T_*x*_. In addition to accommodating a high sulfur loading and buffering the typical volume expansion of sulfur cathodes (from S to Li_2_S), the mesoporous carbon layer can promote the transport of electrolyte ions, resulting in a high rate capability. More importantly, the porous interlayer space improves the exposure of Ti_3_C_2_T_*x*_ to the electrolyte, promoting the effective absorption of polysulfides and their transformation kinetics into Li_2_S_2_/Li_2_S; this mechanism decreases the polysulfides shuttling effect. This solution-based methodology can also be extended to graphene, exfoliated layered compounds (e.g., K_4_Nb_6_O_17_•3H_2_O and MoS_2_ nanosheets), and 2D-shaped nanostructures with various compositions  (e.g., metal organic frameworks, MoO_3_, TiO_2_) to prepare a wide range of 2D heterostructures for different applications such as Li- and Na-ion batteries.^10–13,17^

Considerations related to electrochemical configuration, control over the architecture, dimensionality, and porosity of the mesoporous layers also deserve research attention (Fig. 1b). Zhao et al. reported the construction of a 2D porous heterostructure of graphene uniformly wrapped with OMC by assembling resol-F127 (RF) monomicelles on a graphene aerogel (GA).^14^ The orientation of the mesopores is influenced by the interactions between the 2D nanosheets and the resol-F127 monomicelles. By increasing the RF/GA mass ratio during the assembly process, the aligned mesopore arrays can be controlled and changed from vertical to horizontal. Electrochemical tests indicate that mesoporous carbon with vertical mesopore arrays performs well for supercapacitors constructed with two facing electrodes, while mesoporous carbon containing horizontal mesopore arrays delivers higher power density in microsupercapacitors with an in-plane film-like configuration.^13,15^ A study by Feng et al. showed evidence that the pore sizes of polypyrrole layers on graphene oxide (mPPy@GO) nanosheets could be tuned by selecting block polymers with different block lengths.^11^ When used as supercapacitor electrodes, the mPPy@GO electrodes demonstrated much higher capacitances than the PPy@GO electrodes without pores. In addition, the mPPy@GO electrode with a pore size of 19.3 nm exhibited a better rate and cycling performance than those with pore sizes of 5.8 and 13.2 nm, highlighting the advantages of a large pore size in facilitating ion transport and accommodating volumetric changes during the charge/discharge process. Other porogens, such as SiO_2_, can be added during the assembly of block polymers and precursors to adjust the pore structures of mesoporous monolayers to further optimize their capacity and rate performance.^18^

## Perspectives on the future design and application of 2D materials

Merging 2D materials with monolayered mesoporous structures has introduced a new paradigm to the field of 2D materials and produces unique characteristics that are not found in other 2D hybrid materials, such as the high exposure of their surface area to electrolytes and their well-defined pores for charge transfer. Control over the geometry of the mesoporous layers (e.g., pore size, orientation and layer thickness) can be achieved by selecting different structure-directing agents (e.g., type and molecular weight) and by controlling the assembly conditions to satisfy design requirements. The efficacy and versatility of this concept is demonstrated by the substantially enhanced capacities, improved rate capabilities, and longer life stabilities of energy storage devices, including supercapacitors, lithium–sulfur batteries, Li-ion batteries, and Na-ion batteries. Looking ahead, more in-depth studies of 2D porous heterostructure materials will be needed before their practical application can be developed.

Upcoming challenges faced by 2D porous heterostructures include the exploration of functional mesoporous materials to combine with 2D nanosheets while preserving the stability and integrity of the resulting 2D hybrid. The changes in the physical properties of 2D nanosheets (i.e., surface reconstruction and built-in potential) after forming 2D porous heterostructures have not yet been widely investigated and will undoubtedly be the subjects of future studies. Another goal is to extend the range of porous systems. For example, 2D nanosheets can be covered with more electrochemically active metal-based compounds such as CeO_2_, TiO_2_, MnO_2_, and Mo_2_C to generate novel 2D porous heterostructures. More precise control over the characteristics of the mesoporous layer, such as the number of layers and their thickness (Fig. 1c1), is desirable. Furthermore, new types of 2D nanosheets, such as Janus nanosheets demonstrating distinct properties on each face, can be coated with different mesoporous layers, thereby leading to completely new composites (Fig. 1c2). ‘Machine learning’ can be combined with inorganic synthesis methods to accelerate the optimization of synthetic parameters for the design of target materials and to create superior combination designs with unique properties that surpass current technologies. A genome for 2D porous heterostructures for energy-related applications can be built.

Regarding applications in electrochemical energy storage devices, challenges remain to fully understand the relationship between the reaction kinetics and 2D porous heterostructures (e.g., morphology and electrical properties), which is required to enable critical insights into approaches for designing more sophisticated electrodes. Moreover, the industry-level performance of 2D porous heterostructures for energy-storage devices has yet to be determined. In addition, compared to pure 2D nanosheet electrodes, 2D porous heterostructured electrodes show a lower packing density due to the porous interlayer space, which will lead to a decrease in volumetric capacitance.^19^ One key advantage of 2D heterostructured materials is that they can be processed into freestanding membranes with mechanical flexibility, which can be used directly as electrodes or functional separators. Employing freestanding electrodes without inactive materials (e.g., binders, conducting additives, and current collectors) can improve the gravimetric performance to a certain degree and compensate for the loss in volumetric performance (due to a lower packing density). Advanced processing techniques (e.g., spray coating, 3D printing, electrodeposition, and ink-jet printing) can be employed to manufacture advanced devices for commodity applications.
